# 11-[1-(4-Meth­oxy­phen­yl)-4-oxo-3-phen­oxy­azetidin-2-yl]-14-methyl-12-oxa-8,14-diaza­tetra­cyclo­[8.3.3.0^1,10^.0^2,7^]hexa­deca-2(7),3,5-triene-9,13-dione

**DOI:** 10.1107/S160053681202733X

**Published:** 2012-06-23

**Authors:** S. Sundaramoorthy, R. Rajesh, R. Raghunathan, D. Velmurugan

**Affiliations:** a Centre of Advanced Study in Crystallography and Biophysics, University of Madras, Guindy Campus, Chennai 600 025, India; b Department of Organic Chemistry, University of Madras, Guindy Campus, Chennai - 600 025, India

## Abstract

In the title compound, C_30_H_27_N_3_O_6_, the furan and pyrrolidine rings adopt envelope conformations (with C and N atoms as the flaps, respectively). The piperidine ring is in a distorted boat conformation. The β-lactam ring is planar [maximum deviation = 0.0044 (16) Å] and forms dihedral angles of 30.61 (9) and 85.51 (9)°, respectively, with the attached meth­oxy­phenyl and phen­oxy rings. The crystal packing is stabilized by N—H⋯O and C—H⋯O inter­actions forming *R*_2_^2^(8), *R*_2_^2^(20) and *R*_2_^2^(14) ring motifs. The crystal structure is further consolidated by weak C—H⋯π inter­actions.

## Related literature

For general background to β-lactams, see: Jones *et al.* (1989 ▶); Mehta *et al.* (2010 ▶); Brakhage (1998 ▶). For a related structure, see: Arun *et al.* (2003 ▶). For hydrogen-bond motifs, see: Bernstein *et al.* (1995 ▶).
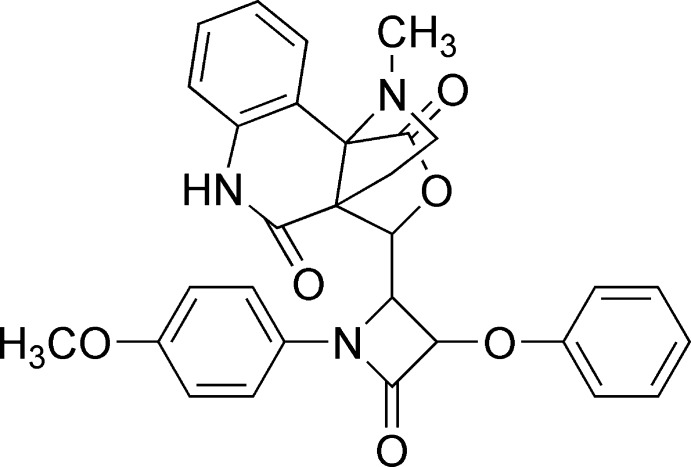


## Experimental

### 

#### Crystal data

C_30_H_27_N_3_O_6_
*M* *_r_* = 525.55
Triclinic, 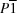

*a* = 10.8510 (4) Å
*b* = 11.1669 (4) Å
*c* = 11.2736 (4) Å
α = 103.087 (2)°
β = 97.367 (2)°
γ = 93.402 (2)°
*V* = 1314.05 (8) Å^3^
*Z* = 2
Mo *K*α radiation
μ = 0.09 mm^−1^
*T* = 293 K
0.25 × 0.23 × 0.2 mm

#### Data collection

Bruker SMART APEXII area-detector diffractometer
Absorption correction: multi-scan (*SADABS*; Bruker, 2008 ▶) *T*_min_ = 0.977, *T*_max_ = 0.981
24049 measured reflections
6513 independent reflections
4678 reflections with *I* > 2σ(*I*)
*R*_int_ = 0.027

#### Refinement

*R*[*F* ^2^ > 2σ(*F* ^2^)] = 0.042
*wR*(*F* ^2^) = 0.114
*S* = 1.03
6513 reflections
354 parameters
H-atom parameters constrained
Δρ_max_ = 0.20 e Å^−3^
Δρ_min_ = −0.19 e Å^−3^

### 

Data collection: *APEX2* (Bruker, 2008 ▶); cell refinement: *SAINT* (Bruker, 2008 ▶); data reduction: *SAINT*; program(s) used to solve structure: *SHELXS97* (Sheldrick, 2008 ▶); program(s) used to refine structure: *SHELXL97* (Sheldrick, 2008 ▶); molecular graphics: *ORTEP-3* (Farrugia, 1997 ▶); software used to prepare material for publication: *SHELXL97* and *PLATON* (Spek, 2009 ▶).

## Supplementary Material

Crystal structure: contains datablock(s) global, I. DOI: 10.1107/S160053681202733X/pv2559sup1.cif

Structure factors: contains datablock(s) I. DOI: 10.1107/S160053681202733X/pv2559Isup2.hkl

Supplementary material file. DOI: 10.1107/S160053681202733X/pv2559Isup3.cml

Additional supplementary materials:  crystallographic information; 3D view; checkCIF report

## Figures and Tables

**Table 1:** Hydrogen-bond geometry (Å, °) *Cg*5 and *Cg*7 are the centroids of the C1–C6 and C22–C27 rings, respectively.

*D*—H⋯*A*	*D*—H	H⋯*A*	*D*⋯*A*	*D*—H⋯*A*
N2—H2*A*⋯O6^i^	0.86	1.99	2.8534 (14)	177
C12—H12⋯O5^ii^	0.93	2.50	3.4245 (17)	171
C16—H16⋯O2^iii^	0.93	2.52	3.3057 (19)	142
C5—H5⋯*Cg*7^iv^	0.93	2.93	3.6523 (18)	136
C25—H25⋯*Cg*5^v^	0.93	2.86	3.5633 (18)	134

Symmetry codes: (i) 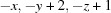; (ii) 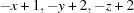; (iii) 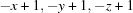; (iv) 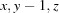; (v) 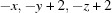.

**Fig. 1.**
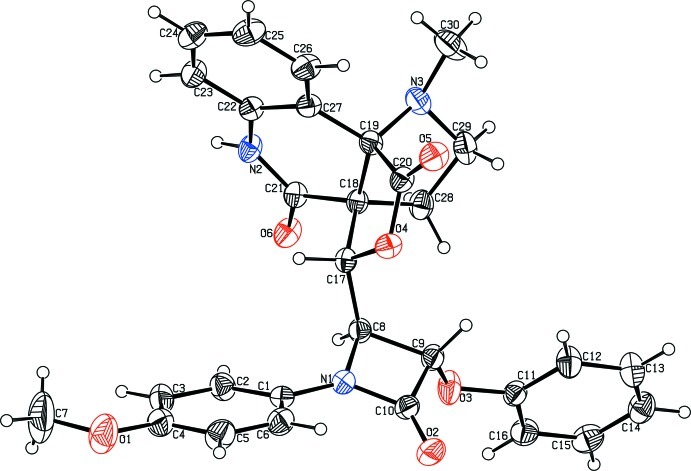
A perspective view of the molecule showing the thermal ellipsoids drawn at the 30% probability level.

**Fig. 2.**
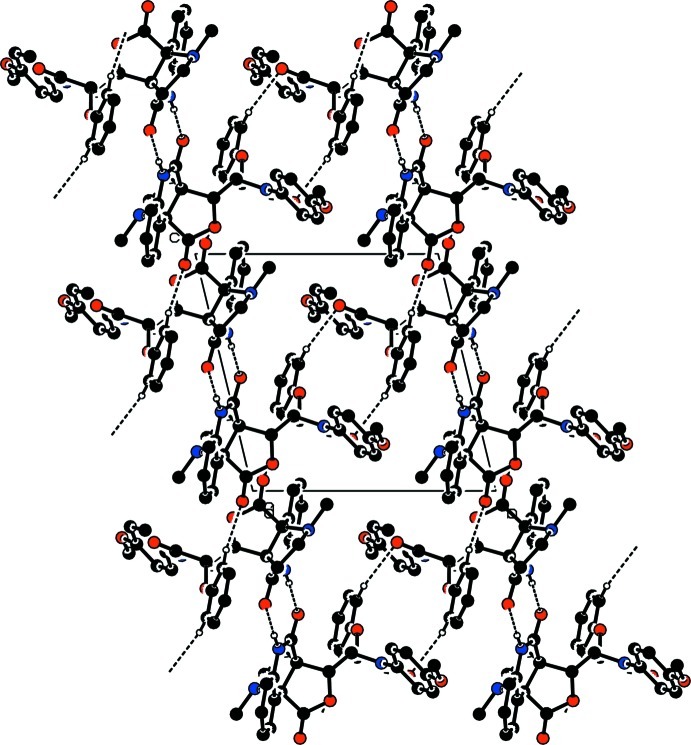
N—H···O and C—H···O interactions (dotted lines) in the crystal structure of the title compound. The crystal packing of the molecules is viewed down the a axis.

## supplementary crystallographic information

### Comment

β-Lactam based antibiotics have been successfully used in the treatment of infectious diseases for many years (Jones *et al.*, 1989). The biological activity of β-lactams is mostly believed to be associated with the chemical reactivity of their β-lactam ring and its substituents, especially at the nitrogen of the 2-azetidinone ring (Mehta *et al.*, 2010). The most commonly used β-lactam antibiotics for the therapy of infectious diseases are penicillin and cephalosporins (Brakhage, 1998). In view of potential applications, the crystal structure determination of the title β-lactam derivative was carried out which is reported in this article.

In the title molecule (Fig. 1), the β-lactam ring makes dihedral angles 30.61 (9)° and 85.51 (9)°, respectively, with the attached methoxyphenyl and phenoxy rings. The furan (O4/C17/C18/C19/C20) and pyrrolidine (C19/C18/C28/C29/N3) rings adopt envelope conformation with C18 and N3 atoms deviating by -0.2044 (13) Å and 0.2529 (13) Å, respectively, from the planes formed by the remaining atoms of the rings. The pyridine ring adopts a distorted boat conformation with atoms C19 and N2 deviating by 0.0932 (12) Å and 0.1741 (14) Å, respectively, from the least-squares plane defined by the remaining atoms (C18/C21/C22/C27) in the ring. The bond lengths and bond angles in the title compound agree with the corresponding bond lengths and angles reported for a closely related compound (Arun *et al.*, (2003).

The crystal packing is stabilized by N—H···O, C—H···O interactions and further consolidated by weak C—H···π interaction. H-atoms bonded to N2, C12 and C16 are involved in hydrogen bonding with atoms O6, O5 and O2 (Fig 2. and Table 1) which connect the molecules forming cyclic centrosymmetric dimers in graph set motifs: *R_2_^2^*(8), *R_2_^2^*(20) and *R_2_^2^*(14), respectively, (Bernstein *et al.*, 1995).

### Experimental

A mixture of methyl 2-(hydroxy(1-(4-methoxyphenyl)-4-oxo-3-phenoxyazetidin -2-yl)methyl)acrylate (1 mmol), isatin (1 mmol) and sarcosine (1 mmol) was refluxed in methanol until completion of the reaction was evidenced by TLC analysis. After completion of the reaction the solvent was evaporated under reduced pressure. The reaction mixture was dissolved in ethyl acetate and washed with water followed by brine solution. The organic layer was separated and evaporated under reduced pressure. The crude mixture was purified by column chromatography using ethyl acetate and hexane as eluent (4: 6). The product was dissolved in chloroform and heated for two minutes. The resulting solution was subjected to crystallization by slow evaporation of the solvent for 48 h resulting in the formation of single crystals.

### Refinement

The H atoms were positioned geometrically with N—H = 0.86 Å and C—H = 0.93, 0.96, 0.97 and 0.98 Å for aryl, methyl, methylene and methyne H atoms, respectively, and constrained to ride on their parent atoms, with *U*_iso_(H) = 1.5*U*_eq_(methyl C) or 1.2*U*_eq_(non-methyl C/N).

### Crystal data

**Table:**

C_30_H_27_N_3_O_6_	*Z* = 2
*M**_r_* = 525.55	*F*(000) = 552
Triclinic, *P*1	*D*_x_ = 1.328 Mg m^−^^3^
Hall symbol: -P 1	Mo *K*α radiation, λ = 0.71073 Å
*a* = 10.8510 (4) Å	Cell parameters from 1025 reflections
*b* = 11.1669 (4) Å	θ = 1.9–28.4°
*c* = 11.2736 (4) Å	µ = 0.09 mm^−^^1^
α = 103.087 (2)°	*T* = 293 K
β = 97.367 (2)°	Block, colourless
γ = 93.402 (2)°	0.25 × 0.23 × 0.2 mm
*V* = 1314.05 (8) Å^3^	

### Data collection

**Table:**

Bruker SMART APEXII area-detector diffractometer	6513 independent reflections
Radiation source: fine-focus sealed tube	4678 reflections with *I* > 2σ(*I*)
Graphite monochromator	*R*_int_ = 0.027
ω and φ scans	θ_max_ = 28.4°, θ_min_ = 1.9°
Absorption correction: multi-scan (*SADABS*; Bruker, 2008)	*h* = −14→14
*T*_min_ = 0.977, *T*_max_ = 0.981	*k* = −14→14
24049 measured reflections	*l* = −14→14

### Refinement

**Table:**

Refinement on *F*^2^	Primary atom site location: structure-invariant direct methods
Least-squares matrix: full	Secondary atom site location: difference Fourier map
*R*[*F*^2^ > 2σ(*F*^2^)] = 0.042	Hydrogen site location: inferred from neighbouring sites
*wR*(*F*^2^) = 0.114	H-atom parameters constrained
*S* = 1.03	*w* = 1/[σ^2^(*F*_o_^2^) + (0.0516*P*)^2^ + 0.1891*P*] where *P* = (*F*_o_^2^ + 2*F*_c_^2^)/3
6513 reflections	(Δ/σ)_max_ < 0.001
354 parameters	Δρ_max_ = 0.20 e Å^−^^3^
0 restraints	Δρ_min_ = −0.19 e Å^−^^3^

### Special details

**Table:**

Geometry. All e.s.d.'s (except the e.s.d. in the dihedral angle between two l.s. planes) are estimated using the full covariance matrix. The cell e.s.d.'s are taken into account individually in the estimation of e.s.d.'s in distances, angles and torsion angles; correlations between e.s.d.'s in cell parameters are only used when they are defined by crystal symmetry. An approximate (isotropic) treatment of cell e.s.d.'s is used for estimating e.s.d.'s involving l.s. planes.
Refinement. Refinement of *F*^2^ against ALL reflections. The weighted *R*-factor *wR* and goodness of fit *S* are based on *F*^2^, conventional *R*-factors *R* are based on *F*, with *F* set to zero for negative *F*^2^. The threshold expression of *F*^2^ > σ(*F*^2^) is used only for calculating *R*-factors(gt) *etc*. and is not relevant to the choice of reflections for refinement. *R*-factors based on *F*^2^ are statistically about twice as large as those based on *F*, and *R*- factors based on ALL data will be even larger.

### Fractional atomic coordinates and isotropic or equivalent isotropic displacement parameters (Å^2^)

**Table:**

	*x*	*y*	*z*	*U*_iso_*/*U*_eq_	
C1	0.16075 (12)	0.58171 (11)	0.74291 (12)	0.0414 (3)	
C2	0.05204 (13)	0.58690 (13)	0.66728 (13)	0.0498 (3)	
H2	0.0538	0.6283	0.6045	0.060*	
C3	−0.05986 (14)	0.53120 (14)	0.68355 (15)	0.0577 (4)	
H3	−0.1331	0.5359	0.6326	0.069*	
C4	−0.06221 (15)	0.46879 (14)	0.77544 (17)	0.0597 (4)	
C5	0.04776 (16)	0.46016 (15)	0.84873 (16)	0.0629 (4)	
H5	0.0468	0.4151	0.9086	0.075*	
C6	0.15848 (14)	0.51742 (14)	0.83404 (14)	0.0530 (4)	
H6	0.2316	0.5129	0.8852	0.064*	
C7	−0.2835 (2)	0.4281 (3)	0.7371 (3)	0.1326 (11)	
H7A	−0.2902	0.5137	0.7385	0.199*	
H7B	−0.3496	0.3981	0.7748	0.199*	
H7C	−0.2896	0.3816	0.6534	0.199*	
C8	0.29483 (11)	0.75643 (12)	0.68381 (12)	0.0399 (3)	
H8	0.2613	0.7452	0.5967	0.048*	
C9	0.43518 (12)	0.73721 (13)	0.70216 (12)	0.0443 (3)	
H9	0.4866	0.8022	0.7653	0.053*	
C10	0.39587 (13)	0.62241 (13)	0.74624 (13)	0.0473 (3)	
C11	0.60370 (12)	0.73217 (12)	0.57939 (12)	0.0423 (3)	
C12	0.69196 (14)	0.79959 (15)	0.67328 (13)	0.0548 (4)	
H12	0.6707	0.8311	0.7509	0.066*	
C13	0.81251 (15)	0.81965 (17)	0.65024 (15)	0.0615 (4)	
H13	0.8727	0.8647	0.7133	0.074*	
C14	0.84512 (15)	0.77452 (15)	0.53627 (15)	0.0575 (4)	
H14	0.9265	0.7894	0.5219	0.069*	
C15	0.75673 (16)	0.70726 (15)	0.44355 (15)	0.0585 (4)	
H15	0.7782	0.6764	0.3659	0.070*	
C16	0.63631 (14)	0.68513 (14)	0.46480 (13)	0.0531 (4)	
H16	0.5770	0.6385	0.4019	0.064*	
C17	0.24857 (11)	0.86746 (11)	0.76324 (11)	0.0364 (3)	
H17	0.1607	0.8472	0.7686	0.044*	
C18	0.25947 (11)	0.99100 (11)	0.72499 (11)	0.0356 (3)	
C19	0.26498 (12)	1.08545 (11)	0.84898 (11)	0.0381 (3)	
C20	0.32569 (12)	1.01372 (12)	0.94040 (11)	0.0405 (3)	
C21	0.14812 (12)	0.99217 (12)	0.62907 (11)	0.0389 (3)	
C22	0.03803 (12)	1.10370 (12)	0.78916 (11)	0.0396 (3)	
C23	−0.07869 (13)	1.13688 (14)	0.81541 (14)	0.0505 (3)	
H23	−0.1453	1.1260	0.7521	0.061*	
C24	−0.09565 (15)	1.18579 (14)	0.93505 (14)	0.0565 (4)	
H24	−0.1736	1.2083	0.9527	0.068*	
C25	0.00299 (16)	1.20127 (15)	1.02851 (14)	0.0588 (4)	
H25	−0.0083	1.2345	1.1094	0.071*	
C26	0.11868 (15)	1.16760 (14)	1.00262 (13)	0.0521 (4)	
H26	0.1843	1.1773	1.0667	0.062*	
C27	0.13889 (12)	1.11940 (11)	0.88234 (11)	0.0397 (3)	
C28	0.38190 (13)	1.02492 (14)	0.67733 (13)	0.0486 (3)	
H28A	0.4339	0.9566	0.6685	0.058*	
H28B	0.3637	1.0459	0.5984	0.058*	
C29	0.44595 (13)	1.13530 (15)	0.77436 (14)	0.0560 (4)	
H29A	0.5067	1.1098	0.8325	0.067*	
H29B	0.4877	1.1933	0.7370	0.067*	
C30	0.38670 (19)	1.29116 (15)	0.94274 (17)	0.0732 (5)	
H30A	0.4438	1.2627	1.0004	0.110*	
H30B	0.3161	1.3193	0.9808	0.110*	
H30C	0.4278	1.3579	0.9178	0.110*	
N1	0.27315 (10)	0.64267 (10)	0.72704 (11)	0.0449 (3)	
N3	0.34465 (11)	1.18995 (10)	0.83485 (11)	0.0490 (3)	
N2	0.05128 (10)	1.05094 (10)	0.66654 (9)	0.0434 (3)	
H2A	−0.0082	1.0570	0.6106	0.052*	
O1	−0.16778 (12)	0.41466 (13)	0.80246 (16)	0.0936 (5)	
O2	0.44842 (10)	0.54240 (10)	0.78242 (11)	0.0653 (3)	
O3	0.47929 (9)	0.70977 (11)	0.58733 (9)	0.0612 (3)	
O4	0.31803 (9)	0.89176 (8)	0.88606 (8)	0.0428 (2)	
O5	0.37457 (10)	1.05310 (9)	1.04423 (8)	0.0543 (3)	
O6	0.14758 (9)	0.93721 (10)	0.52118 (8)	0.0522 (3)	

### Atomic displacement parameters (Å^2^)

**Table:**

	*U*^11^	*U*^22^	*U*^33^	*U*^12^	*U*^13^	*U*^23^
C1	0.0402 (7)	0.0347 (6)	0.0461 (7)	0.0049 (5)	0.0049 (5)	0.0033 (5)
C2	0.0469 (8)	0.0514 (8)	0.0494 (8)	0.0007 (6)	−0.0001 (6)	0.0133 (6)
C3	0.0420 (8)	0.0585 (9)	0.0689 (10)	−0.0005 (6)	−0.0037 (7)	0.0156 (8)
C4	0.0478 (9)	0.0514 (9)	0.0829 (12)	0.0033 (7)	0.0138 (8)	0.0197 (8)
C5	0.0595 (10)	0.0636 (10)	0.0767 (11)	0.0118 (8)	0.0168 (8)	0.0343 (9)
C6	0.0495 (8)	0.0543 (8)	0.0571 (9)	0.0110 (6)	0.0026 (7)	0.0182 (7)
C7	0.0458 (12)	0.159 (3)	0.215 (3)	−0.0101 (13)	0.0112 (15)	0.098 (2)
C8	0.0348 (6)	0.0435 (7)	0.0391 (7)	0.0028 (5)	0.0031 (5)	0.0066 (5)
C9	0.0343 (7)	0.0528 (8)	0.0395 (7)	0.0053 (5)	0.0040 (5)	−0.0016 (6)
C10	0.0411 (7)	0.0456 (7)	0.0481 (8)	0.0099 (6)	−0.0006 (6)	−0.0011 (6)
C11	0.0371 (7)	0.0481 (7)	0.0402 (7)	0.0080 (5)	0.0063 (5)	0.0061 (6)
C12	0.0449 (8)	0.0751 (10)	0.0389 (7)	−0.0007 (7)	0.0069 (6)	0.0032 (7)
C13	0.0448 (8)	0.0806 (11)	0.0557 (9)	−0.0065 (7)	0.0035 (7)	0.0143 (8)
C14	0.0469 (8)	0.0686 (10)	0.0665 (10)	0.0082 (7)	0.0194 (7)	0.0286 (8)
C15	0.0659 (10)	0.0638 (9)	0.0511 (9)	0.0167 (8)	0.0259 (8)	0.0121 (7)
C16	0.0533 (9)	0.0575 (9)	0.0431 (8)	0.0090 (7)	0.0088 (6)	−0.0014 (6)
C17	0.0321 (6)	0.0424 (7)	0.0346 (6)	0.0030 (5)	0.0032 (5)	0.0103 (5)
C18	0.0327 (6)	0.0420 (6)	0.0324 (6)	0.0035 (5)	0.0029 (5)	0.0106 (5)
C19	0.0394 (7)	0.0397 (6)	0.0333 (6)	0.0025 (5)	−0.0017 (5)	0.0087 (5)
C20	0.0391 (7)	0.0478 (7)	0.0348 (7)	0.0077 (5)	0.0037 (5)	0.0103 (5)
C21	0.0389 (7)	0.0459 (7)	0.0325 (6)	0.0037 (5)	0.0031 (5)	0.0120 (5)
C22	0.0419 (7)	0.0410 (7)	0.0369 (7)	0.0065 (5)	0.0058 (5)	0.0104 (5)
C23	0.0431 (8)	0.0585 (9)	0.0500 (8)	0.0111 (6)	0.0065 (6)	0.0113 (7)
C24	0.0541 (9)	0.0602 (9)	0.0597 (9)	0.0180 (7)	0.0205 (7)	0.0132 (7)
C25	0.0733 (11)	0.0616 (9)	0.0439 (8)	0.0214 (8)	0.0195 (8)	0.0072 (7)
C26	0.0608 (9)	0.0548 (8)	0.0378 (7)	0.0156 (7)	0.0025 (6)	0.0053 (6)
C27	0.0440 (7)	0.0389 (6)	0.0354 (7)	0.0077 (5)	0.0030 (5)	0.0080 (5)
C28	0.0401 (7)	0.0591 (9)	0.0515 (8)	0.0019 (6)	0.0118 (6)	0.0212 (7)
C29	0.0402 (8)	0.0667 (10)	0.0622 (9)	−0.0083 (7)	−0.0005 (7)	0.0256 (8)
C30	0.0839 (13)	0.0511 (9)	0.0714 (11)	−0.0157 (8)	−0.0125 (9)	0.0062 (8)
N1	0.0376 (6)	0.0405 (6)	0.0550 (7)	0.0062 (4)	0.0021 (5)	0.0097 (5)
N3	0.0484 (7)	0.0442 (6)	0.0508 (7)	−0.0058 (5)	−0.0042 (5)	0.0127 (5)
N2	0.0374 (6)	0.0595 (7)	0.0325 (6)	0.0108 (5)	−0.0003 (4)	0.0107 (5)
O1	0.0535 (7)	0.0957 (10)	0.1521 (14)	0.0036 (7)	0.0264 (8)	0.0661 (10)
O2	0.0554 (6)	0.0561 (6)	0.0787 (8)	0.0190 (5)	−0.0064 (5)	0.0097 (6)
O3	0.0361 (5)	0.0928 (8)	0.0415 (5)	0.0004 (5)	0.0060 (4)	−0.0101 (5)
O4	0.0497 (5)	0.0452 (5)	0.0346 (5)	0.0108 (4)	0.0027 (4)	0.0119 (4)
O5	0.0599 (6)	0.0642 (6)	0.0340 (5)	0.0152 (5)	−0.0064 (4)	0.0066 (4)
O6	0.0505 (6)	0.0716 (7)	0.0321 (5)	0.0171 (5)	0.0013 (4)	0.0071 (5)

### Geometric parameters (Å, º)

**Table:**

C1—C2	1.3763 (19)	C16—H16	0.9300
C1—C6	1.3819 (19)	C17—O4	1.4480 (14)
C1—N1	1.4127 (17)	C17—C18	1.5382 (17)
C2—C3	1.384 (2)	C17—H17	0.9800
C2—H2	0.9300	C18—C21	1.5174 (17)
C3—C4	1.375 (2)	C18—C19	1.5406 (17)
C3—H3	0.9300	C18—C28	1.5532 (17)
C4—O1	1.3643 (19)	C19—N3	1.4610 (17)
C4—C5	1.383 (2)	C19—C27	1.5119 (18)
C5—C6	1.374 (2)	C19—C20	1.5491 (17)
C5—H5	0.9300	C20—O5	1.1937 (15)
C6—H6	0.9300	C20—O4	1.3538 (16)
C7—O1	1.406 (3)	C21—O6	1.2313 (15)
C7—H7A	0.9600	C21—N2	1.3354 (16)
C7—H7B	0.9600	C22—C27	1.3896 (18)
C7—H7C	0.9600	C22—C23	1.3897 (18)
C8—N1	1.4769 (17)	C22—N2	1.4039 (16)
C8—C17	1.5091 (17)	C23—C24	1.376 (2)
C8—C9	1.5447 (18)	C23—H23	0.9300
C8—H8	0.9800	C24—C25	1.376 (2)
C9—O3	1.4127 (16)	C24—H24	0.9300
C9—C10	1.533 (2)	C25—C26	1.379 (2)
C9—H9	0.9800	C25—H25	0.9300
C10—O2	1.2065 (16)	C26—C27	1.3916 (18)
C10—N1	1.3621 (17)	C26—H26	0.9300
C11—O3	1.3762 (16)	C28—C29	1.514 (2)
C11—C12	1.3776 (19)	C28—H28A	0.9700
C11—C16	1.3791 (18)	C28—H28B	0.9700
C12—C13	1.381 (2)	C29—N3	1.4591 (19)
C12—H12	0.9300	C29—H29A	0.9700
C13—C14	1.369 (2)	C29—H29B	0.9700
C13—H13	0.9300	C30—N3	1.463 (2)
C14—C15	1.371 (2)	C30—H30A	0.9600
C14—H14	0.9300	C30—H30B	0.9600
C15—C16	1.376 (2)	C30—H30C	0.9600
C15—H15	0.9300	N2—H2A	0.8600
			
C2—C1—C6	119.55 (13)	C17—C18—C19	102.07 (9)
C2—C1—N1	119.70 (12)	C21—C18—C28	109.79 (10)
C6—C1—N1	120.75 (12)	C17—C18—C28	117.05 (10)
C1—C2—C3	120.73 (14)	C19—C18—C28	104.08 (10)
C1—C2—H2	119.6	N3—C19—C27	113.49 (11)
C3—C2—H2	119.6	N3—C19—C18	103.26 (10)
C4—C3—C2	119.61 (14)	C27—C19—C18	114.36 (10)
C4—C3—H3	120.2	N3—C19—C20	114.43 (10)
C2—C3—H3	120.2	C27—C19—C20	109.15 (10)
O1—C4—C3	124.43 (15)	C18—C19—C20	101.56 (10)
O1—C4—C5	115.97 (15)	O5—C20—O4	121.28 (12)
C3—C4—C5	119.59 (15)	O5—C20—C19	128.54 (12)
C6—C5—C4	120.75 (15)	O4—C20—C19	110.18 (10)
C6—C5—H5	119.6	O6—C21—N2	122.05 (11)
C4—C5—H5	119.6	O6—C21—C18	120.01 (11)
C5—C6—C1	119.72 (14)	N2—C21—C18	117.91 (11)
C5—C6—H6	120.1	C27—C22—C23	120.91 (12)
C1—C6—H6	120.1	C27—C22—N2	120.55 (11)
O1—C7—H7A	109.5	C23—C22—N2	118.52 (12)
O1—C7—H7B	109.5	C24—C23—C22	120.00 (14)
H7A—C7—H7B	109.5	C24—C23—H23	120.0
O1—C7—H7C	109.5	C22—C23—H23	120.0
H7A—C7—H7C	109.5	C25—C24—C23	119.86 (14)
H7B—C7—H7C	109.5	C25—C24—H24	120.1
N1—C8—C17	112.75 (10)	C23—C24—H24	120.1
N1—C8—C9	86.95 (10)	C24—C25—C26	120.18 (14)
C17—C8—C9	119.27 (11)	C24—C25—H25	119.9
N1—C8—H8	111.8	C26—C25—H25	119.9
C17—C8—H8	111.8	C25—C26—C27	121.19 (14)
C9—C8—H8	111.8	C25—C26—H26	119.4
O3—C9—C10	113.64 (11)	C27—C26—H26	119.4
O3—C9—C8	110.17 (11)	C22—C27—C26	117.85 (12)
C10—C9—C8	86.06 (10)	C22—C27—C19	119.04 (11)
O3—C9—H9	114.6	C26—C27—C19	123.10 (12)
C10—C9—H9	114.6	C29—C28—C18	104.97 (11)
C8—C9—H9	114.6	C29—C28—H28A	110.8
O2—C10—N1	132.35 (15)	C18—C28—H28A	110.8
O2—C10—C9	136.01 (13)	C29—C28—H28B	110.8
N1—C10—C9	91.63 (10)	C18—C28—H28B	110.8
O3—C11—C12	125.27 (12)	H28A—C28—H28B	108.8
O3—C11—C16	114.56 (12)	N3—C29—C28	104.19 (11)
C12—C11—C16	120.11 (13)	N3—C29—H29A	110.9
C11—C12—C13	118.92 (13)	C28—C29—H29A	110.9
C11—C12—H12	120.5	N3—C29—H29B	110.9
C13—C12—H12	120.5	C28—C29—H29B	110.9
C14—C13—C12	121.25 (15)	H29A—C29—H29B	108.9
C14—C13—H13	119.4	N3—C30—H30A	109.5
C12—C13—H13	119.4	N3—C30—H30B	109.5
C13—C14—C15	119.39 (15)	H30A—C30—H30B	109.5
C13—C14—H14	120.3	N3—C30—H30C	109.5
C15—C14—H14	120.3	H30A—C30—H30C	109.5
C14—C15—C16	120.33 (14)	H30B—C30—H30C	109.5
C14—C15—H15	119.8	C10—N1—C1	134.48 (12)
C16—C15—H15	119.8	C10—N1—C8	95.34 (10)
C15—C16—C11	119.98 (14)	C1—N1—C8	130.16 (10)
C15—C16—H16	120.0	C29—N3—C19	105.18 (11)
C11—C16—H16	120.0	C29—N3—C30	113.88 (13)
O4—C17—C8	108.95 (10)	C19—N3—C30	118.63 (12)
O4—C17—C18	105.16 (9)	C21—N2—C22	125.78 (11)
C8—C17—C18	118.21 (10)	C21—N2—H2A	117.1
O4—C17—H17	108.0	C22—N2—H2A	117.1
C8—C17—H17	108.0	C4—O1—C7	118.60 (16)
C18—C17—H17	108.0	C11—O3—C9	120.94 (10)
C21—C18—C17	108.20 (10)	C20—O4—C17	110.29 (9)
C21—C18—C19	115.74 (10)		
			
C6—C1—C2—C3	1.7 (2)	C28—C18—C21—N2	−132.92 (12)
N1—C1—C2—C3	−177.96 (13)	C27—C22—C23—C24	−0.1 (2)
C1—C2—C3—C4	−0.7 (2)	N2—C22—C23—C24	178.28 (13)
C2—C3—C4—O1	177.42 (15)	C22—C23—C24—C25	−0.3 (2)
C2—C3—C4—C5	−1.4 (2)	C23—C24—C25—C26	−0.2 (2)
O1—C4—C5—C6	−176.28 (15)	C24—C25—C26—C27	1.0 (2)
C3—C4—C5—C6	2.7 (3)	C23—C22—C27—C26	0.9 (2)
C4—C5—C6—C1	−1.7 (2)	N2—C22—C27—C26	−177.45 (12)
C2—C1—C6—C5	−0.4 (2)	C23—C22—C27—C19	−178.23 (12)
N1—C1—C6—C5	179.18 (13)	N2—C22—C27—C19	3.39 (18)
N1—C8—C9—O3	113.13 (12)	C25—C26—C27—C22	−1.4 (2)
C17—C8—C9—O3	−132.40 (12)	C25—C26—C27—C19	177.76 (13)
N1—C8—C9—C10	−0.63 (9)	N3—C19—C27—C22	94.95 (14)
C17—C8—C9—C10	113.83 (12)	C18—C19—C27—C22	−23.17 (16)
O3—C9—C10—O2	69.4 (2)	C20—C19—C27—C22	−136.13 (12)
C8—C9—C10—O2	179.68 (17)	N3—C19—C27—C26	−84.17 (15)
O3—C9—C10—N1	−109.63 (12)	C18—C19—C27—C26	157.71 (12)
C8—C9—C10—N1	0.68 (10)	C20—C19—C27—C26	44.75 (17)
O3—C11—C12—C13	−176.86 (15)	C21—C18—C28—C29	123.53 (12)
C16—C11—C12—C13	0.4 (2)	C17—C18—C28—C29	−112.67 (12)
C11—C12—C13—C14	0.4 (3)	C19—C18—C28—C29	−0.95 (13)
C12—C13—C14—C15	−0.6 (3)	C18—C28—C29—N3	−24.05 (14)
C13—C14—C15—C16	−0.1 (2)	O2—C10—N1—C1	1.2 (3)
C14—C15—C16—C11	0.9 (2)	C9—C10—N1—C1	−179.72 (14)
O3—C11—C16—C15	176.51 (14)	O2—C10—N1—C8	−179.77 (16)
C12—C11—C16—C15	−1.1 (2)	C9—C10—N1—C8	−0.72 (10)
N1—C8—C17—O4	63.06 (13)	C2—C1—N1—C10	−150.00 (15)
C9—C8—C17—O4	−36.66 (15)	C6—C1—N1—C10	30.4 (2)
N1—C8—C17—C18	−177.08 (10)	C2—C1—N1—C8	31.3 (2)
C9—C8—C17—C18	83.20 (15)	C6—C1—N1—C8	−148.33 (13)
O4—C17—C18—C21	−154.91 (9)	C17—C8—N1—C10	−119.86 (11)
C8—C17—C18—C21	83.28 (13)	C9—C8—N1—C10	0.71 (10)
O4—C17—C18—C19	−32.37 (11)	C17—C8—N1—C1	59.21 (17)
C8—C17—C18—C19	−154.18 (10)	C9—C8—N1—C1	179.78 (13)
O4—C17—C18—C28	80.49 (12)	C28—C29—N3—C19	41.75 (13)
C8—C17—C18—C28	−41.32 (15)	C28—C29—N3—C30	173.28 (12)
C21—C18—C19—N3	−95.07 (12)	C27—C19—N3—C29	−166.39 (11)
C17—C18—C19—N3	147.70 (10)	C18—C19—N3—C29	−42.03 (12)
C28—C18—C19—N3	25.49 (12)	C20—C19—N3—C29	67.44 (13)
C21—C18—C19—C27	28.72 (15)	C27—C19—N3—C30	64.87 (16)
C17—C18—C19—C27	−88.51 (11)	C18—C19—N3—C30	−170.77 (13)
C28—C18—C19—C27	149.28 (10)	C20—C19—N3—C30	−61.31 (17)
C21—C18—C19—C20	146.12 (10)	O6—C21—N2—C22	172.34 (12)
C17—C18—C19—C20	28.89 (11)	C18—C21—N2—C22	−5.68 (19)
C28—C18—C19—C20	−93.32 (11)	C27—C22—N2—C21	12.6 (2)
N3—C19—C20—O5	52.09 (19)	C23—C22—N2—C21	−165.80 (13)
C27—C19—C20—O5	−76.31 (17)	C3—C4—O1—C7	−3.4 (3)
C18—C19—C20—O5	162.59 (14)	C5—C4—O1—C7	175.4 (2)
N3—C19—C20—O4	−127.41 (12)	C12—C11—O3—C9	−11.3 (2)
C27—C19—C20—O4	104.19 (12)	C16—C11—O3—C9	171.25 (13)
C18—C19—C20—O4	−16.92 (13)	C10—C9—O3—C11	−109.08 (14)
C17—C18—C21—O6	−79.81 (14)	C8—C9—O3—C11	156.28 (12)
C19—C18—C21—O6	166.44 (11)	O5—C20—O4—C17	176.75 (12)
C28—C18—C21—O6	49.02 (16)	C19—C20—O4—C17	−3.70 (13)
C17—C18—C21—N2	98.26 (13)	C8—C17—O4—C20	150.77 (10)
C19—C18—C21—N2	−15.49 (16)	C18—C17—O4—C20	23.12 (12)

### Hydrogen-bond geometry (Å, º)

*Cg*5 and *Cg*7 are the centroids of the C1–C6 and C22–C27 rings, respectively.

**Table:**

*D*—H···*A*	*D*—H	H···*A*	*D*···*A*	*D*—H···*A*
N2—H2*A*···O6^i^	0.86	1.99	2.8534 (14)	177
C12—H12···O5^ii^	0.93	2.50	3.4245 (17)	171
C16—H16···O2^iii^	0.93	2.52	3.3057 (19)	142
C26—H26···O5	0.93	2.56	3.150 (2)	122
C5—H5···*Cg*7^iv^	0.93	2.93	3.6523 (18)	136
C25—H25···*Cg*5^v^	0.93	2.86	3.5633 (18)	134

Symmetry codes: (i) −*x*, −*y*+2, −*z*+1; (ii) −*x*+1, −*y*+2, −*z*+2; (iii) −*x*+1, −*y*+1, −*z*+1; (iv) *x*, *y*−1, *z*; (v) −*x*, −*y*+2, −*z*+2.
